# Tracheal polypoid combined small cell lung cancer (C‐SCLC): A case report

**DOI:** 10.1111/1759-7714.13992

**Published:** 2021-05-14

**Authors:** Alberto Testori, Giorgio Ferraroli, Camilla De Carlo, Paola Bossi, Marco Alloisio, Giuseppe Mangiameli

**Affiliations:** ^1^ Division of Thoracic Surgery IRCCS Humanitas Research Hospital Milan Italy; ^2^ Department of Pathology Humanitas Clinical and Research Center – IRCCS Rozzano Italy; ^3^ Department of Biomedical Sciences Humanitas University Milan Italy

**Keywords:** cSCLC, SCLC, tracheal tumor

## Abstract

Small cell lung cancer (SCLC) is an aggressive malignancy with a poor prognosis that accounts for 10% of all cases of clinical lung cancer. Due to its high growth fraction and rapid doubling time it is usually diagnosed as extensive local or metastatic disease in 60%–70% of cases. Combined small cell lung cancer (C‐SCLC) is a relatively rare subtype of SCLC and is defined as SCLC combined with any elements of non‐small cell lung cancer (NSCLC). Clinical presentation of SCLC as an isolated pedunculated endotracheal lesion is an especially rare occurrence. Here, we report for the first time the occurrence of a C‐SCLC as a polypoid tumor of the trachea diagnosed in an 80‐year‐old woman admitted to the emergency department with a principal complaint of cough and wheezing.

## Graphical abstract

Small cell lung cancer (SCLC) is an aggressive malignancy with a poor prognosis that accounts for 10% of all clinical lung cancer. Due to its high growth fraction and rapid doubling time it is usually diagnosed as extensive local or metastatic disease in 60%–70% of cases. Combined small cell lung cancer (C‐SCLC) is a relatively rare subtype of SCLC and is defined as SCLC combined with any elements of non‐small cell lung cancer (NSCLC). Clinical presentation of SCLC as an isolated pedunculated endotracheal lesion is a rare occurrence. Here, we report for the first time the occurrence of a C‐SCLC as a polypoid tumor of the trachea diagnosed in an 80‐year‐old woman admitted with a principal complaint of cough and wheezing.

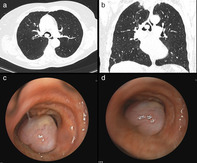

## INTRODUCTION

Small cell lung cancer (SCLC) is prognostically the worst cancer among malignant neoplasm of the lung. It accounts for 10% of all clinically diagnosed cases of lung cancer.^1^ Most SCLCs are pure SCLC (P‐SCLC), while some can be combined with additional components of any histological types of non‐small cell lung cancer (NSCLC), and has been defined as combined small cell lung cancer (C‐SCLC) according to the World Health Organization (WHO) in 2001.^2^

As a consequence of its aggressive behavior (estimated doubling time of 30–60 days), SCLC is usually diagnosed (60%–70%) as extensive local or metastatic^3^ Both primary or metastatic localisation to the trachea are an especially rare occurrence.

Here, we report for the first time a case of a C‐SCLC which presented as an isolated polypoid pedunculated endotracheal lesion with a thin implantation base diagnosed in a patient admitted to the emergency department with a principal complaint of cough and wheezing.

## CASE REPORT

An 80‐year‐old woman with an active smoking history of >50‐pack year and without a significant previous medical history was admitted to the emergency department with dyspnea of progressive appearance which had worsened in the last 48 h.

**FIGURE 1:**
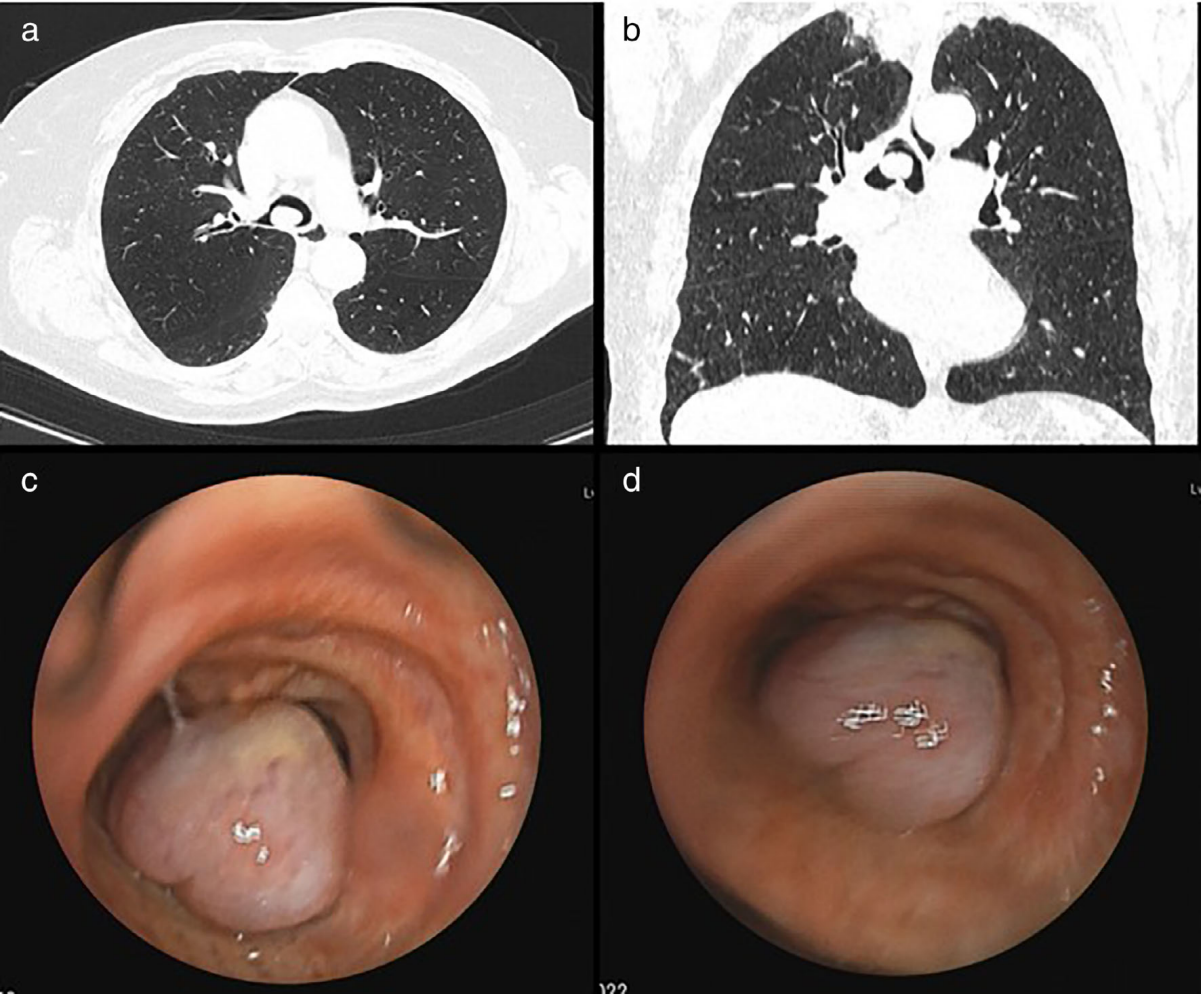
Axial and coronal contrast computed tomography (CT) (a, b) and endoscopic view during rigid broncoscopy (c, d) showing the presence of a tracheal pedunculated polyp lesion with a thin implantation base on the carina

A chest computed tomography (CT) scan showed the presence of a vegetative tracheal lesion measuring 15 × 12 × 6 mm originating from the posterior wall of the trachea above the carina (Figure 1(a) and (b)). A rigid bronchoscopy revealed a pedunculated, glossy and lobulated lesion with a thin implantation base on the carina, alternatively obstructing either the right or left main bronchus during breathing and protruding to the trachea (Figure 1(c) and (d)). The lesion was completely removed using a laser to the base of the lesion. The procedure and postoperative course were uneventful. The patient experienced immediate relief of symptoms and was discharged on postoperative day one.

The final pathologist report revealed the presence of a poorly differentiated carcinoma, with morphophenotypic features consistent with a C‐SCLC presenting a squamous cell component (pT1N0M0).

**FIGURE 2:**
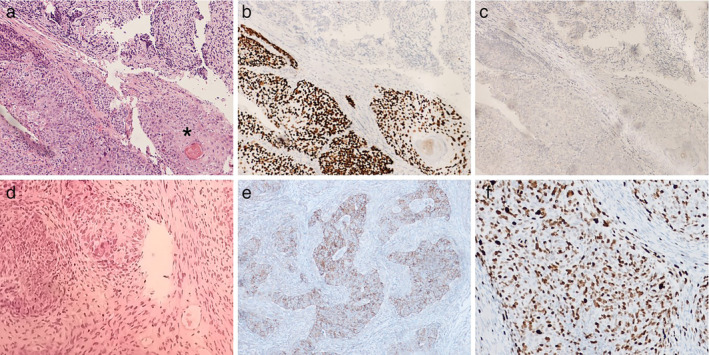
Immunohistochemistry (IHC) of the tracheal lesion in the case reported here (10x magnification) allowed small cell lung cancer (SCLC) to be distinguished from non‐small cell lung cancer (NSCLC) components. (a) Hematoxylin & eosin (H&E) showing a poorly differentiated, solid proliferation exhibiting a squamous component with variable keratinisation degree (keratin pearl, see asterisk). (b) Same field as (a) showing p40 positive nuclear staining as a standard marker of squamous differentiation. (c) Same field as (a) showing NUT negative nuclear staining. (d) H&E taken from another field of the same tumor with a prominent neuroendocrine area. (e) Same field as (b) with positive cytoplasmic staining for synaptophysin, as typical neuroendocrine markers. (f) Ki‐67 brown nuclear staining

**FIGURE 3:**
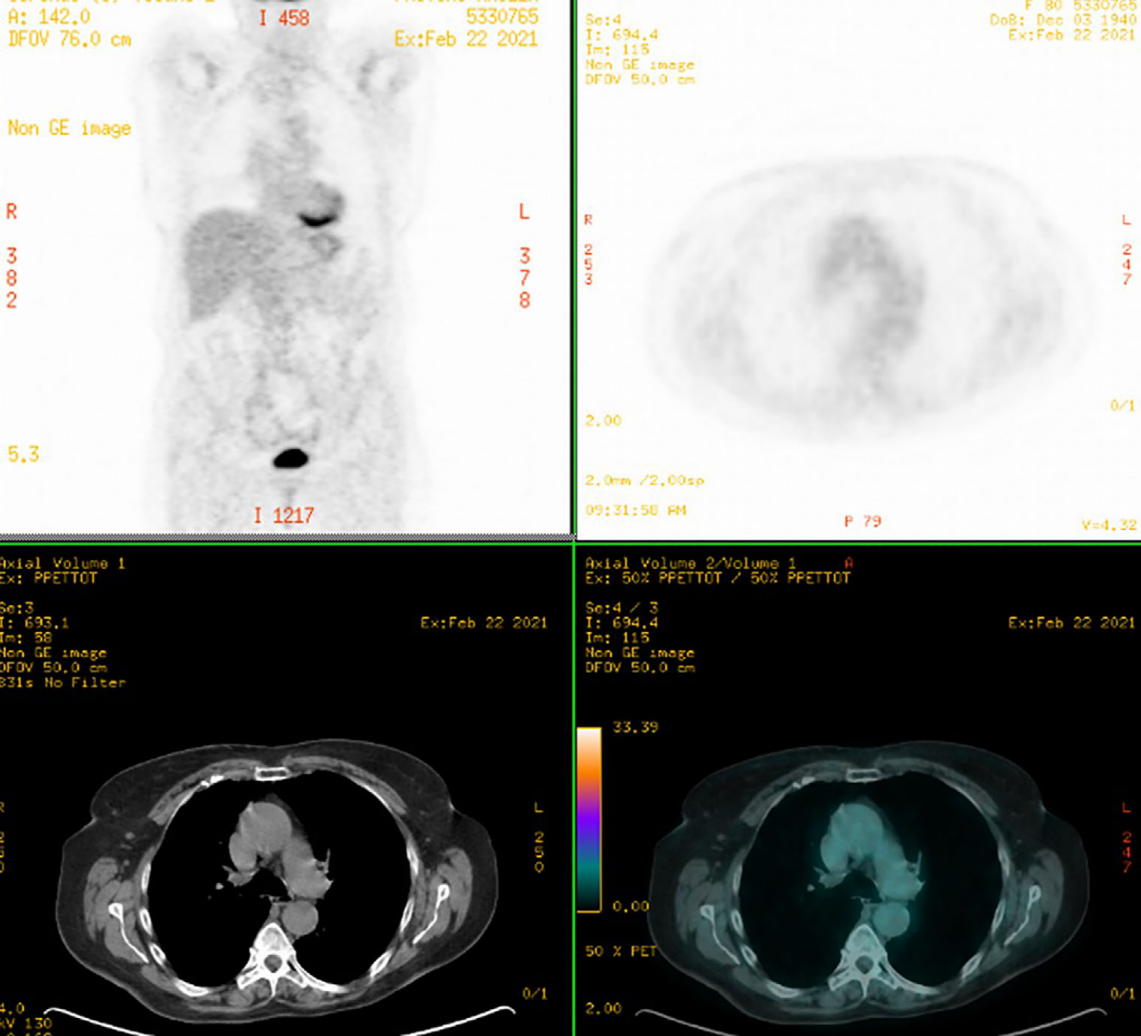
18F‐fluorodeoxyglucose (FDG) positron emission tomography (PET) performed for clinical staging did not show local or distant pathological uptake

SCLC and NSCLC components were not clearly demarcated, but it was possible to clearly distinguish them following immunohistochemistry (IHC). In fact, the morphological neuroendocrine component was strongly positive for typical neuroendocrine markers such as synaptophysin (Figure 2(e)). However, the squamous pattern was positive for p40 (Figure 2(b)), a standard marker of squamous differentiation. The negativity for NUT excluded NUT carcinoma, an aggressive neoplasm defined by translocations involving the NUT gene and squamous differentiation, frequently involving the head and neck.^4^ The neuroendocrine component showed a very high ki‐67 index (Figure 2(f)), reaching 80% of the neoplastic cells. PDL1 was <1%. An 18F‐fluorodeoxyglucose (FDG) positron emission tomography (PET) scan performed three weeks later for clinical staging purposes did not show pathological tracheal residual metabolic uptake (Figure 3).

Following a multidisciplinary board discussion, the patient was scheduled for three cycles of etoposide and cisplatin (EP) adjuvant chemotherapy.

## DISCUSSION

Here, we present for the first time a patient with C‐SCLC which presented as an isolated polypoid pedunculated endotracheal lesion with a thin implantation base on the carina.

Primary tracheal carcinoma is an uncommon malignancy of the respiratory tract, accounting for 0.1% of all such tumors. Squamous cell carcinoma is the most common histology seen in primary tracheal carcinoma (60%–90%). Other described histologies include adenoid cystic carcinoma, carcinoid, adenocarcinoma, carcinosarcoma, mucoepidermoid carcinoma, chondrosarcoma and small cell carcinoma.^5^

C‐SCLC is a relatively rare subtype of SCLC and is defined as SCLC combined with any elements of NSCLC. The exact mechanisms and histogenesis of C‐SCLC remain unclear. Biologically, the same cancer stem cell could differentiate in morphologically distinct constituents.^6^ The combined NSCLC histological types include adenocarcinoma, squamous cell carcinoma, large cell carcinoma, large cell neuroendocrine carcinoma, or any other rare component, such as giant cell carcinoma or sarcoma‐like cancer, etc. Incidence of C‐SCLC has been previously reported to range from 2% to 28% in different studies.^7^ C‐SCLCs are typically treated according to SCLC regimens for extensive‐stage cancer, even if systemic chemotherapy appears to have a lower efficacy on C‐SCLC. A recent study reports that the prognosis of C‐SCLCs did not significantly differ from that of P‐SCLCs after surgical resection.^7^

As mentioned above, the tracheal occurrence for both primitive and metastatic SCLC is rare. SCLC commonly metastasizes to the mediastinum, liver, bone, adrenals, and the brain but secondary endotracheal metastasis is an especially rare occurrence. Until now, only four cases have been described in the literature.^8^

Similarly, SCLC is a rare primary tracheal malignancy. In a recent review, less than 80 cases of tracheal SCLC were described.^9^ Interestingly, only two cases reported a tracheal SCLC which presented as a sessile nonresectable tracheal polypoid lesion. Jain et al. reported SCLC presenting as a tracheal polyp attached circumferentially to the tracheal wall with left paratracheal extension without evidence of any lung parenchymal pathology or mediastinal adenopathy.^10^ Heikal reported a case of SCLC presenting as small sessile polyp in the proximal trachea about 3 to 4 cm below the true vocal cords.^9^ We have not found in literature similar case reports of tracheal pedunculated polypoid SCLC.

With regard to histology, only three cases of tracheal C‐SCLC have been reported to date in the literature. Manigil et al. reported a primary carcinoma of the trachea with combined features of SCLC, SCC, and giant‐cell carcinoma.^11^ In 40 years experience of treating uncommon primary tracheal tumors, Gaissart et al. reported five cases of SCLC where two had mixed features with SCC pathology.^12^

In conclusion, this is the first report of a case of C‐SCLC which presented as an isolated polypoid pedunculated endotracheal lesion with a thin implantation base submitted to a R0 resection in rigid bronchoscopy. R0 resection was a “surprise” after an emergency procedure performed with a diagnostic and therapeutic finality. Management guidelines for such rare malignancies are not optimally defined. Decision‐making should take into account prior treatment and needs to be individualized.

## CONFLICT OF INTEREST

The authors declare that there is no conflict of interest.
